# Case report: A cyclic neutropenia patient with *ELANE* mutation accompanied by hemophagocytic lymphohistiocytosis

**DOI:** 10.3389/fimmu.2024.1474429

**Published:** 2024-11-29

**Authors:** Lang Yu, Yulin Li, Wenhui Li, Yishi Zhang, Wenli He, Xuemei Tang, Yunfei An, Xiaodong Zhao

**Affiliations:** ^1^ National Clinical Research Center for Child Health and Disorders, Ministry of Education Key Laboratory of Child Development and Disorders, Children’s Hospital of Chongqing Medical University, Chongqing, China; ^2^ Chongqing Key Laboratory of Child Rare Diseases in Infection and Immunity, Children’s Hospital of Chongqing Medical University, Chongqing, China; ^3^ Molecular Medicine Diagnostic and Testing Center, Chongqing Medical University, Chongqing, China; ^4^ Department of Rheumatology & Immunology, Children’s Hospital of Chongqing Medical University, Chongqing, China

**Keywords:** cyclic congenital neutropenia, CYN, SCN, hemophagocytic lymphohistiocytosis, HLH

## Abstract

Many inborn errors of immunity may accompany secondary hemophagocytic lymphohistiocytosis (HLH), a condition typically characterized by impaired cytotoxic T and NK cell function. A considerable proportion of HLH cases also stem from chronic granulomatosis with phagocytic dysfunction. However, the development of secondary HLH in patients with severe congenital neutropenia (SCN) or cyclic neutropenia (CyN) with abnormal phagocytic cell counts has been less frequently reported. Herein, we present a case of a pediatric patient with *ELANE* mutation-associated CyN who developed HLH subsequent to severe bacterial, fungal, and viral infections. Notable observations included impaired NK cell degranulation function (CD107a). To the best of our knowledge, this represents the first documented instance of HLH in patients with CyN attributed to an *ELANE* mutation. Thus, our study establishes a link between ELANE-related CyN and HLH, underscoring the importance of considering HLH as a potential complication in these patients.

## Introduction

Severe congenital neutropenia (SCN) is a rare genetic disorder characterized by a lack of mature neutrophils in the bone marrow, an absolute neutrophil count (ANC) of less than 0.5 × 10^9^/L in peripheral blood, and potentially life-threatening early systemic bacterial infection in infants (1). More than half of SCN cases have mutations in the neutrophil elastase (NE), encoded by the *ELANE* gene. In addition, *ELANE* mutations can also lead to cyclic neutropenia (CyN), characterized by regular fluctuations in peripheral blood neutrophil count, typically occurring on a 21-day cycle (2).

Hemophagocytic lymphohistiocytosis (HLH) presents as a syndrome characterized by severe systemic inflammation. Its hallmark features encompass persistent fever, diminished blood cell counts, hepatosplenomegaly, and elevated classical HLH biomarkers (3, 4). Various genetic disorders associated with HLH are categorized under familial HLH, which includes PRF1 mutations, pathological alterations in *UNC13D*, *STX11*, and *STXBP2*. Additionally, HLH emerges as a prevalent manifestation in several other genetic disorders, such as certain pigmentary conditions, X-linked lymphoproliferative disease, Epstein-Barr virus (EBV) susceptibility disorders, specific *CDC42* mutations, and *NLRC4* activation mutations. Collectively, these conditions are referred to as hereditary HLH disorders (5–7). Moreover, numerous primary immunodeficiency (PID) diseases seldom accompany HLH, typically observed in the context of infection, leading to aberrant macrophage activation-induced excessive inflammation (8, 9). In such instances, HLH is classified as secondary HLH. Notably, congenital neutropenia cases associated with diminished phagocytic cell counts are rarely reported to be associated with HLH development.

Here, we described a patient with CyN who developed HLH and central nervous system complications after suffering from severe invasive bacterial, fungal, and viral infections. Our report establishes a connection between CyN associated with ELANE mutations and HLH, thereby broadening the disease spectrum of this condition.

## Method

### Genetic diagnosis

Peripheral blood samples from the patients were sent to MyGenostics (Beijing, China) for Whole-exome sequencing (WES) (10, 11). DNA extraction was performed from whole blood using the Whole Blood DNA Extraction Kit (BIOTEKE DP1802). Mutations in the *ELANE* gene were confirmed by Sanger sequencing. The primers for the PCR: *ELANE* Forward primers: CGGGCTAATCCACGGAATTGC, Reverse primers: ATGCTGGAGAGTGTGGGTGTG.

### NK cell function analysis

For the detection of CD107a expression on NK cells, peripheral blood mononuclear cells (PBMCs) previously frozen from patients and healthy controls were thawed and cultured in a 37°C, 5% CO2 incubator for 2 hours. Subsequently, the stimulation group was co-cultured with K562 cells for 4 hours. Following incubation, the cells were retrieved and stained with CD3-Percp (300,326 Biolegend), CD56-PE (318,306 Biolegend), and CD107a-APC (328,620 Biolegend) at room temperature for 30 minutes (12). The stained cells were then analyzed using a flow cytometer.

### Diagnostic guidelines for hemophagocytic lymphohistiocytosis

This article adopts the diagnostic guidelines of HLH-2004 (3), and meets five of the eight criteria to consider diagnosing HLH. The specific content is shown in **Table 1**.

**Table 1:** HLH-2004 diagnostic criteria in this patient.

The diagnosis HLH can be established if one of either 1 or 2 below is fulfilled:	Fulfilled	NO	Not done
(1) A molecular diagnosis consistent with HLH		✓	
(2) Diagnostic criteria for HLH fulfilled (five out of the eight criteria below)	✓		
Fever	1/8		
Splenomegaly			
Cytopenias (affecting ≥ 2 of 3 lineages in the peripheral blood):	2/8		
Hemoglobin < 90 g/L (in infants < 4 weeks: hemoglobin < 100 g/L)			
Platelets < 100 * 109/L			
Neutrophils < 1.0 * 109/L			
Hypertriglyceridemia and/or hypofibrinogenemia	3/8		
Fasting triglycerides ≥ 3.0 mmol/L (i.e., ≥ 265 mg/dl)			
Fibrinogen ≤ 1.5 g/L			
Hemophagocytosis in bone marrow or spleen or lymph nodes. No evidence of malignancy	4/8		
Low or absent NK-cell activity (according to local laboratory reference).	5/8		
Ferritin ≥ 500 mg/L.	6/8		
Soluble CD25 ≥ 2400 U/ml.	7/8		

✓means yes, or have conducted.

## Results

### Case presentation

**Figure 1:**
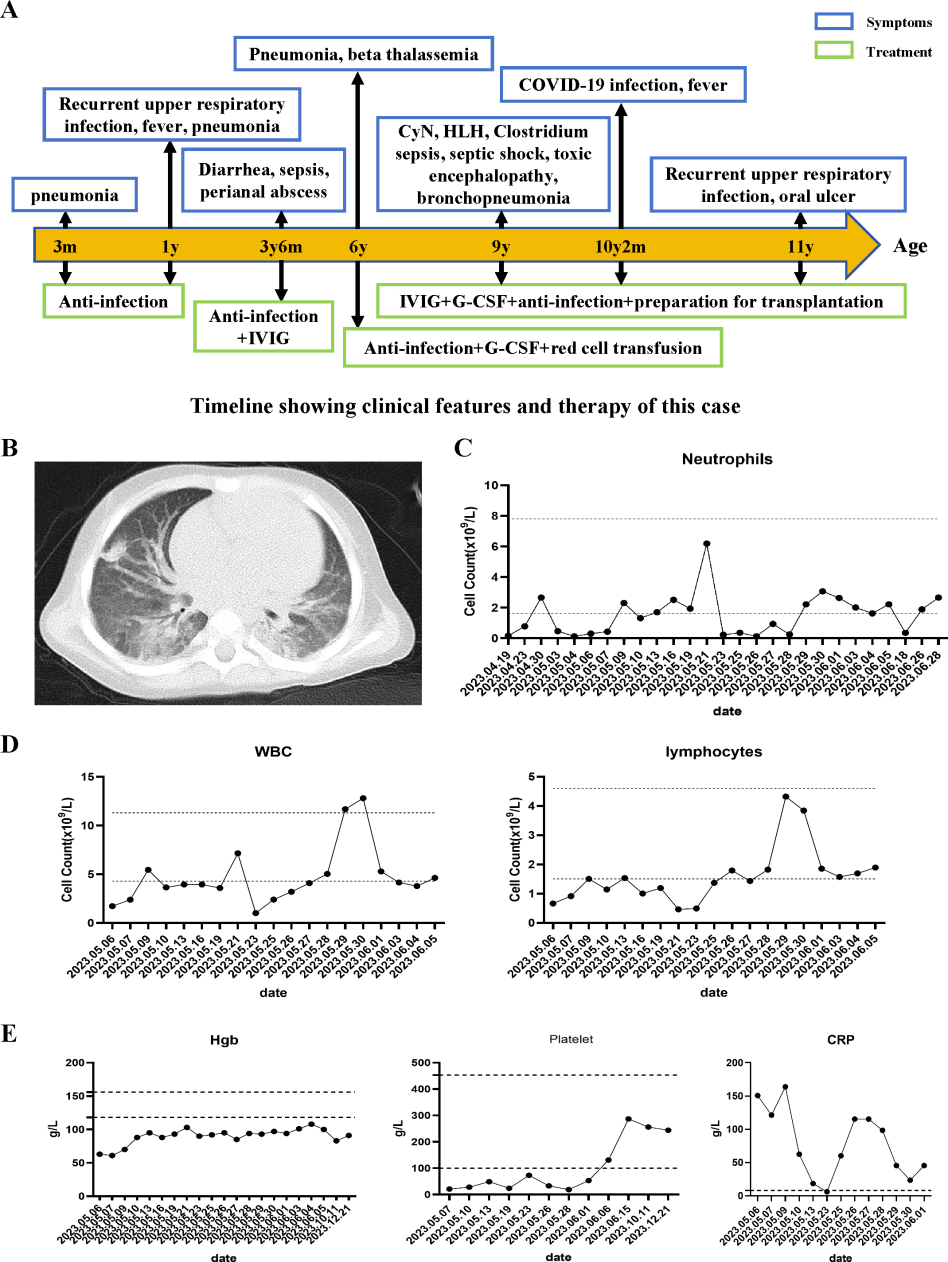
Clinical course and manifestations of the patient. **(A)** Clinical course of the patient. **(B)** Chest CT shows widespread lesions in both lungs. **(C, D)** Neutrophils, WBC, and lymphocytes count of the patient's peripheral blood. **(E)** The levels of hemoglobin, platelets, and C-reactive protein (CRP) during the patient's course.

The patient, a eleven-year-old second child of non-consanguineous parents from China, was referred to our hospital at the age of 9 years following severe sepsis accompanied by septic shock and neutropenia (**Figure 1A**). The patient resided in a favorable living environment and had no significant family history. Clinically, the patient presented with a sustained high fever lasting over 20 days, with temperatures exceeding 40°C, accompanied by cough, expectoration, abdominal pain, and neurological manifestations, including unresponsive breathing, limb tremors, cyanosis of the lips, and urinary incontinence. Neurological examination: The patient presented to our hospital with clear consciousness, poor mental responsiveness, normal cranial nerve reflexes, normal muscle tone, and no signs of meningeal irritation or pathological reflexes (At the time of admission, the patient’s neurological symptoms were already in the recovery phase). Others: Temperature (T) 38.6 (36.0-37.0°C), Respiration rate (RR) 21 (18-20/min), Heart rate (HR) 112 (60-100/min), Blood pressure (BP) 98/58 (90-110/65-75mmHg), Oxygen saturation (SpO2) 98% (95-100%). While intracranial infection was suspected, cranial magnetic resonance imaging (MRI) revealed no apparent abnormalities. However, an abnormal electroencephalogram (EEG) indicated increased background activity during the wakefulness period, which may suggest that the current brain injury is in the recovery phase. Unfortunately, the patient’s family declined lumbar puncture. Chest CT scans depicted bilateral lung inflammation (**Figure 1B**), abdominal CT suggested a possible intestinal perforation, cholecystitis and chronic abscess (from an external hospital).

### Laboratory examination

Despite receiving appropriate antibiotic therapy (initially ampicillin and netilmicin, later switched to meropenem), the patient’s clinical condition continued to deteriorate. In addition to neutropenia, the patient exhibited a progressive decline in hemoglobin levels and platelet counts, accompanied by elevated erythrocyte sedimentation rate and C-reactive protein (CRP) levels (**Figures 1C–E**). In addition, the patient’s triglyceride levels were elevated, fibrinogen levels were normal, transaminase levels were elevated, coagulation time was prolonged, and D-dimer levels were elevated. Cytokine analysis revealed elevated levels of IL-6 (252.16pg/mL), IL-8 (186.46pg/mL), and IL-10 (9.56pg/mL) (**Table 2**). Blood gas analysis: pH 7.466 (7.35-7.45), PO2 84.5 mmHg (80-100 mmHg), PCO2 46.2 mmHg (35-45 mmHg), HCO3 15.6 mmol/L (17-29 mmol/L), BE -7.0 mmol/L (-7.5-4.5 mmol/L), LAC 0.5 mmol/L (0.5-1.7 mmol/L), P/F 437.6 mmHg (400-500 mmHg).

**Table 2:** Laboratory features of patient at diagnosis of HLH.

Laboratory features	Levels at diagnosis	References values
Fibrinogen	3.46	1.22-3.89g/L
Ferritin	**966**	28-365 ng/ml
Triglycerides	**3.64**	0.30-1.80mmo1/L
sCD25	**15533**	78-520 U/mL (> 2400)
D-Dimer	**0.89**	0-0.73 ng/mL
ALT	30.0	0-30.0 U/L
Albumin	*37.7*	39.0-54.0g/L
Total Bilirubin	6.2	0-26.0umo1/L
Direct Bilirubin	2.9	0-10.0umol/L
INR	1.22	0.8-1.5s
LDH	*141.0*	145.0-300.0U/L
IgA	2.51	0.510-2.590 g/L
IgG	14.9	5.280-21.900 g/L
IgM	0.862	0.48-2.26 g/L
IgE unit/L	12.7	0-165 unti/L
C3	1.34	0.7-2.0g/L
C5	0.35	0.11-0.61g/L
IL-16	7.34	0.00-12.4 pg/mL
IL-2	0.57	0.00-9.80 pg/mL
IL-4	0.92	0.00-3.00 pg/mL
IL-5	0.33	0.00-3.10 pg/mL
IL-6	**252.16**	0.00-16.60 pg/mL
IL-8	**186.46**	0.00-20.60 pg/mL
IL-10	**9.56**	0.00-4.90 pg/mL
IL-12p70	1.64	0.00-3.40 pg/mL
IL-17A	0.0	0.00-14.8 pg/mL
TNF-a	0.63	0.00-5.20 pg/mL
IFN-y	0.71	0.00-17.30 pg/mL
IFN-a	0.75	0.00-8.50 pg/mL

IL, interleukin; TNF, tumor necrosis factor; IFN, interferon; bold, increased subpopulation. Italic, decreased subpopulations.

**Figure 2:**
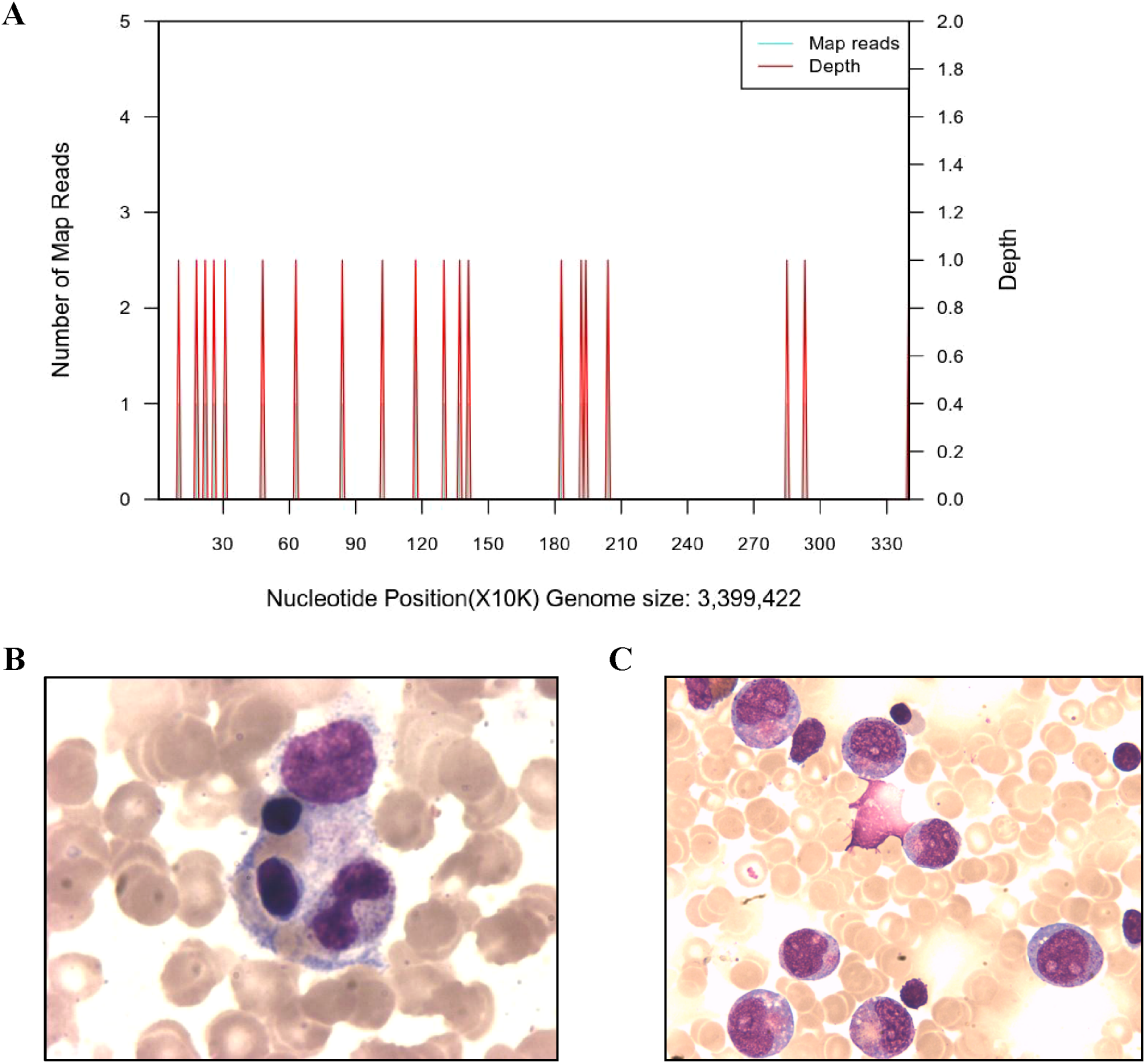
Imaging examination and the Next-generation sequencing of blood. **(A)** Next-generation sequencing of Clostridium septicum in blood. **(B)** Bone marrow aspirates show hemophagocytic cells that engulf neutrophils, platelets, and red blood cells. **(C)** The bone marrow cytology examination of the patient showed early maturation arrest of granulocytes when the neutrophil count remained low after HLH recovery.

**Figure 3:**
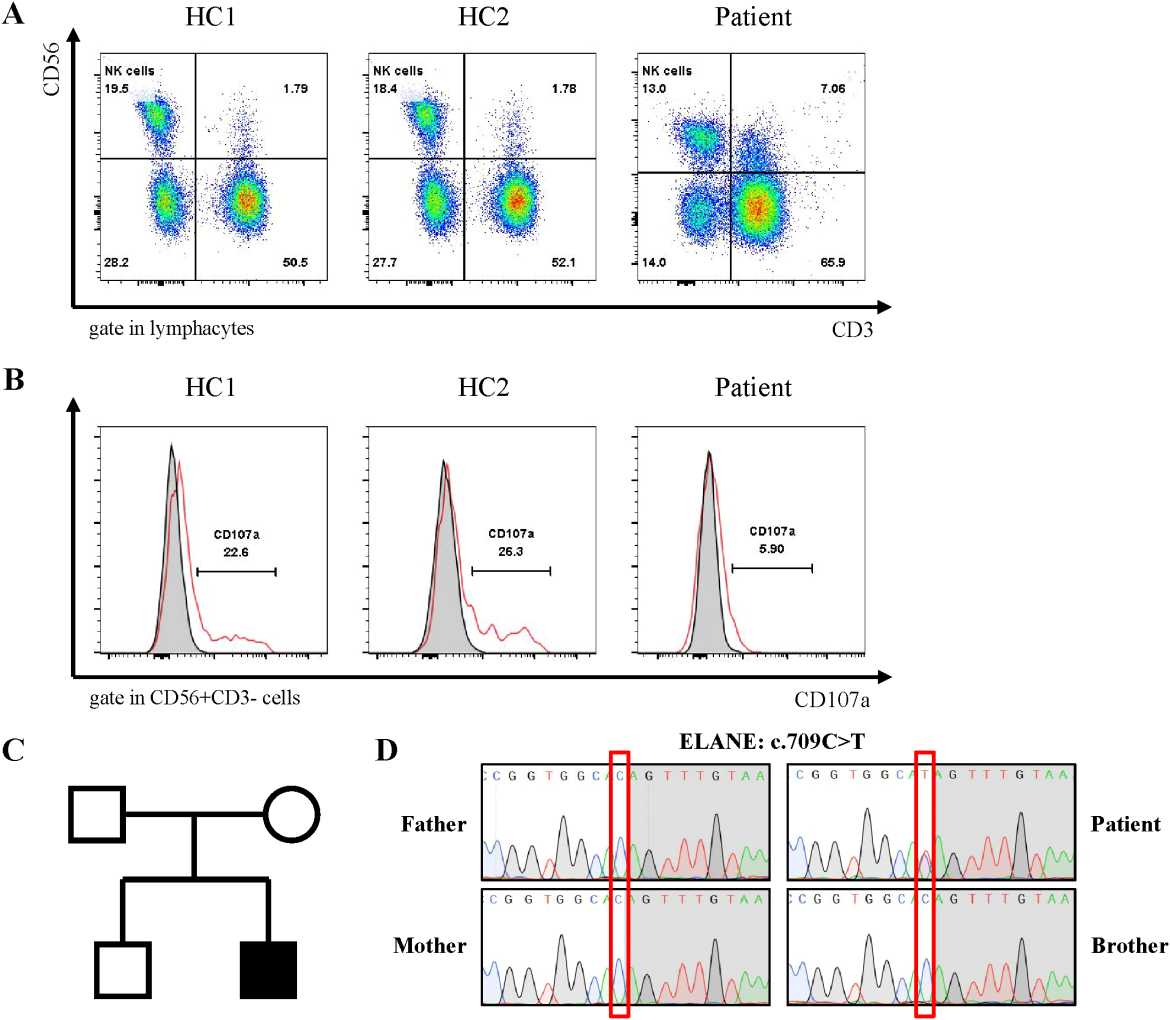
Genetic diagnosis of ELANE gene mutations and Immunological Characteristics of patients. **(A)** Family pedigree. The patient's parents and brother both have normal phenotypes. **(B)** Sanger sequencing confirmed the ELANE mutation (c.709C>T, p.Gln237Ter) in the patient. **(C, D)** Flow cytometry detection of NK cell activity and CD107a analysis of degranulation function.

Despite negative results from blood, urine, and fecal cultures, the patient’s critical condition persisted, accompanied by ongoing deterioration in laboratory parameters, including declining cell counts and prolonged clotting times. Screening for Mycobacterium tuberculosis yielded negative results. Both PCR and mNGS results for nasopharyngeal secretions were negative (**Supplementary Table 3**). Peripheral blood metagenomics (mNGS) testing revealed positive findings for Clostridium perfringens and HSV-1, PCR confirmed this result (HSV-1 1.2*10^4^ copies/ml) (**Figure 2A** **) (** 13). Although fungal elements were not detected, the patient’s serum fungal β-D glucan test remained abnormal. Consequently, the patient’s antibacterial therapy was augmented to include therapeutic antifungal agents. Bone marrow aspiration and biopsy unveiled abnormal phagocytic blood cells engulfing neutrophils, red blood cells, and platelets (**Figure 2B**). Notably, HLH is typically associated with impaired activity and function of cytotoxic T lymphocytes and NK cells (14). Accordingly, we observed a reduction in the number of peripheral blood lymphocytes in the patient. Further analysis revealed a decrease in absolute CD3^+^ T cells, B cells, and NK cells, although the proportions of each subgroup remained relatively unchanged (**Supplementary Table 1**, **Figure 3A**). Additionally, analysis of NK cells indicated diminished NK cell degranulation function (CD107a) compared to the healthy control group (**Figure 3B**). However, in a recent repetition of the experiment, results showed no significant difference in NK cell CD107a degranulation function between the patient and healthy controls (**Supplementary Figure 1**). Moreover, the patient’s serum soluble CD25 (sCD25) level increased (15533U/ml) (**Table 2**). Based on the patient’s clinical presentation and laboratory findings, a diagnosis of HLH was established, as the patient met 7 out of 8 diagnostic criteria for HLH (**Table 1** **) (** 3).

### Genetic diagnosis

The patient underwent whole-exome sequencing, revealing no known hereditary HLH-related mutations. However, a *de novo* variant, c.709C>T (p.Gln237Ter), in exon 5 of the patient’s *ELANE* gene was identified. Neither of the patient’s parents nor his brother carried this variant (**Figures 3C, D**). This variant has been previously reported in patients with SCN (5). Furthermore, heterozygous variants, including c.316-197C>T in the *HBB* gene, were detected in the patient, inherited from the mother, with anticipated effects on exon splicing (**Supplementary Table 2**). These variants have been previously associated with beta-thalassemia (15). Notably, the patient had been diagnosed with beta-thalassemia in an external hospital three years ago (HbA2 5.2%, reference 1.5-3.7%).

### Treatment and outcomes

The patient’s medical history reveals a pattern of recurrent infections and neutropenia (**Figure 1A**). Ongoing monitoring of peripheral blood neutrophil levels indicated a cyclic neutropenia pattern, occurring approximately every three weeks. Notably, these assessments were conducted without regular granulocyte colony-stimulating factor (G-CSF) administration. Additionally, recent bone marrow cytology examination revealed significant granulocyte maturation arrest (**Figure 2C**). Consequently, the patient was diagnosised of CyN (associated with *ELANE* mutation), Clostridium sepsis, septic shock, HLH, toxic encephalopathy, bronchopneumonia, and beta-thalassemia. Following a comprehensive treatment regimen that included antimicrobial therapies (meropenem, vancomycin, metronidazole, piperacillin-tazobactam) along with a 3-day course of methylprednisolone bolus at 20 mg/kg, high-dose intravenous immunoglobulins at 1 g/kg for 2 days, and supportive care with leukocyte-suspension red blood cell infusions at 1 unit per day for 2 days, the patient’s condition improved significantly, ultimately leading to discharge. It is worth noting that the HSV-1 viremia resolved spontaneously without antiviral treatment, despite the administration of high-dose corticosteroids (Confirmed as negative by PCR). The patient received regular infusion of G-CSF to prevent infection, and preparations for transplantation were underway.

## Discussion

HLH represents a rare, hyper-acute, and potentially life-threatening clinical condition resulting from severe disruption of immune homeostasis (5, 6). HLH occurring in individuals bearing known mutations in genes associated with granule-dependent cytotoxicity are classified as familial HLH (FHL). As per the International Union of Immunological Societies (IUIS), FHL is categorized as Inborn Errors of Immunity (IEI) presenting with HLH as the predominant clinical manifestation (16). Conversely, when HLH arises secondary to infections, autoimmune phenomena, or malignancy in the absence of specific mutations in FHL-associated genes, it is termed secondary HLH (7, 8, 17). Compared to primary HLH, the pathogenesis of secondary HLH in primary immunodeficiency remains incompletely elucidated. Previous reports have linked the risk of HLH in these hereditary conditions to immune system dysregulation, resulting in uncontrolled cytokine release, or persistent infections due to protective immune deficiencies (6). While neutrophil dysfunction, such as chronic granulomatosis disease (CGD), contributes significantly to secondary HLH cases, alterations in neutrophil counts are typically associated with congenital neutropenia, with only a few reported cases of HLH (5). CGD is characterized by recurrent infections, heightened inflammation, and excessive cytokine production, potentially rendering patients more prone to HLH during infections (18). Karapinar et al. documented three cases in which patients with *HAX1* mutations were diagnosed with congenital neutropenia following HLH onset (19). However, to date, there have been no reports of HLH in patients with the most common *ELANE* mutation associated with SCN, including CyN. We attribute our patient’s HLH primarily to severe sepsis and the inflammatory cytokine storm induced by multiple infections, including Clostridium septicum, fungi, and viruses.

Clostridium septicum, an anaerobic, motile, spore-producing Gram-positive bacterium, can cause severe infections such as bacteremia, muscle necrosis, and encephalitis through blood transmission, particularly in pediatric patients with neutropenia, leading to severe sepsis (20, 21). In our patient, although no EBV infection was observed, HSV-1 infection was detected with suspected intracranial involvement; however, further cerebrospinal fluid examination was not feasible. While the exact mechanism linking HSV-1 infection to HLH remains unclear, we propose that the concurrent presence of HSV-1, bacterial, and fungal infections may have triggered an excessive cytokine storm, subsequently leading to HLH in this patient (22).

HLH is commonly associated with impaired activity and function of cytotoxic T lymphocytes and NK cells (6). In our patient, we observed a decrease in the absolute number of T cells and NK cells, with the proportions remaining within the normal range, as well as impaired degranulation function of NK cells (CD107a), which was only observed during episodes of HLH. It is currently unclear whether this impairment was related to CyN caused by *ELANE* mutations. Furthermore, impaired NK cell function was previously observed in patients with chronic granulomatous disease during HLH episodes (23). Further research is needed to elucidate the specific mechanisms underlying the impaired NK cell function in SCN/CyN and CGD during HLH, which may be related to severe infections. In addition, we observed mild CD4^+^T lymphocyte reduction in patients during HLH. The results of lymphocyte subset testing conducted at an external hospital were normal. There was no evidence of other immunodeficiencies, and whole exome sequencing (WES) did not identify any additional potential pathogenic gene mutations related to immunodeficiencies. Although the exact cause of lymphocyte depletion remained unclear, we believe this phenomenon may be related to the underlying infection and the course of HLH.

A limitation of this study is the absence of data on serum levels of CXCL19 and IL-18 in patients with HLH, particularly during the early stages of the disease. These cytokines are critical in the pathogenesis of HLH and hold potential value for early diagnosis (24). Additionally, it is noteworthy that while our patients exhibited significantly elevated levels of IL-6 and IL-8, as well as mildly elevated IL-10, their IFN-γ levels remained within the normal range. Therefore, assessing CXCL19 and IL-18 levels could be crucial for improving early diagnostic accuracy in such cases. HLH is a severe inflammatory syndrome caused by abnormally activated macrophages and cytotoxic T cells, with IFN-γ and IL-6 considered key cytokines in the pathogenesis of HLH. Cases of HLH with normal IFN-γ levels have been observed in chronic granulomatous disease and other types of primary immunodeficiencies (PID) (20, 25–27). The occurrence of HLH in patients with normal IFN-γ levels raised an intriguing question. Previous studies suggest that HLH can also be triggered by excessive stimulation of Toll-like receptor 9 (TLR9), leading to overactivation of phagocytic cells and a subsequent cytokine storm (28). In summary, uncovering the underlying mechanisms of these disease processes remains a valuable and challenging area of research.

In addition to the *ELANE* and *HBB* gene mutations already associated with the disease, there were other potential pathogenic mutations identified in the WES results of the patient described in this article, such as *MEFV*. The *MEFV* c.442G>C (p.Glu148Gln) mutation has previously been linked to autosomal dominant familial Mediterranean fever (OMIM: 134610) and acute febrile neutrophilic dermatitis (OMIM: 608068). However, the patient has not yet exhibited symptoms of recurrent fever (outside of the HLH episode) or neutrophilic dermatitis, and the patient’s mother, who carries the same mutation, has not shown these clinical manifestations either. Given that some individuals may present with incomplete clinical phenotypes, we cannot entirely rule out the pathogenicity of this mutation. Other reported pathogenic mutations, such as *CCDC47*, *CEP152*, and *HYDIN*, have been excluded due to the patient’s heterozygous carrier status not aligning with autosomal recessive inheritance patterns and the absence of corresponding clinical features. Additionally, other gene mutations predicted to be of uncertain significance by pathogenicity software are listed in **Supplementary Table 2**. As the patient has not yet exhibited related clinical manifestations, these mutations are beyond the scope of this article.

In summary, we presented a case of cyclic neutropenia in a patient harboring a heterozygous Q237X mutation in the *ELANE* gene, who developed secondary HLH following severe and explosive bacterial, fungal, and viral infections. To the best of our knowledge, this represents the first documented occurrence of secondary HLH in *ELANE*-related congenital neutropenia. Our findings contribute to broadening the phenotype associated with this disease and emphasize the importance of considering HLH diagnosis in patients with hemophagocytic manifestations of both SCN and CyN.

## Patient perspective

The patient and his parents were fully engaged throughout the treatment and told us that his symptoms improved significantly after treatment and remained stable recently. The patient consented to the publication of this case report and written informed consent was obtained.

## Data Availability

The datasets generated during and/or analyzed during the current study are available from the corresponding author on reasonable request.
