# Supplementary material for: Case report: A cyclic neutropenia patient with ELANE mutation accompanied by hemophagocytic lymphohistiocytosis
Source: Front Immunol. 2024 Nov 29;15:1474429. doi: 10.3389/fimmu.2024.1474429 (PMC11638164; doi:10.3389/fimmu.2024.1474429)
Supplement: Supplementary file 1 [file Table1.docx]

**Supplementary materials for “Case Report: A Cyclic Neutropenia Patient with ELANE Mutation Accompanied by Hemophagocytic Lymphohistiocytosis”**

**Supplementary table 1. The immune cell phenotyping of the patient.**

**Lymphocytes**

CD4

CD8

B cells

NK cells

γδ of CD3 T cells

**CD4 subpopulations**

Naive

Terminally differentiated

Central memory

Effector memory

**CD8 subpopulations**

Naive

Terminally differentiated

Central memory

Effector memory

**B cell subpopulation**

Naive B cells

Memory B cells

Transitional B cells

Plasmablasts

CD4/CD8

**Percentage (%)**

**Reference value**

**Reference value**

**Absolute number (cells/ml)**

24.00-38.72

**335.2**

531-1110

*39.9*

480-1112

9.19-19.48

216-536

10.01-26.98

**125.1**

246-792

8.10-20.76

151.6

124-388

**31.7**

39.72-69.59

**106.3**

294-683

0.3

0.10-1.29

1.1

0-9

24.24-52.73

196.1

165-475

9.5

32

24-87

64.8

41.41-73.04

294.8

245-657

2.5

2.01-21.65

**11.3**

12-164

92-287

1.53-15.39

13.3

9-130

8.96-24.09

28-89

**112.9**

123-362

**2.1**

2.50-9.07

7-37

19.0

3-21.0

**0.74**

0.81-1.66

29.4

**454.9**

21.01-33.94

**177.5**

15.57

11.0

18.1

*58.5*

3.40-11.17

13.21-37.89

29.7

135.1

2.9

63.6

51.84-77.61

24.0

42.6

**3.7**

*10.7*

0.70-5.67

Italics, increased subpopulation; bold, decreased subpopulation

**Supplementary table 2.** Other identified variants were found in WES of this patient.

**Gene Chromosomal position Reference transcript Nucleic acid change Amino acid chang Het/hom Prediction**

*HBB*  chr11-5247153 NM_000518.5 c.316-197C>T splicing het Pathogenic

*TRND*  chr6-123687328 NM_006073.4 c.2109G>T p.Gln703His het Likely-Benign

*CEP152* chr15-49059645 NM_014985.4 c.2034T>G p.Tyr678Ter het Pathogenic

*CCDC47* chr17-61829437 NM_020198.6 c.1234C>T p.Arg412Ter het Likely-Pathogenic

*TCF3* chr19-1621888 NM_003200.5 c.904G>A p.Gly302Ser het Uncertain

*MEFV* chr16-3304626 NM_000243.3 c.442G>C p.Glu148Gln het Uncertain

*HYDIN* chr16-70896016-70896016 NM_001270974.2 c.11712del p.Gln3905ArgfsTer5 het Likely-Pathogenic

*CREBBP* chr16-3778613 NM_004380.3 c.6435G>C p.Met2145Ile het Uncertain

*CD46* chr1-207956640 NM_002389.4 c.992A>G p.Tyr331Cys het Uncertain

*SLC3A1* chr2-44507932 NM_000341.4 c.508C>G p.Leu170Val het Uncertain

*DES* chr2-220283249 NM_001927.4 c.65C>G p.Pro22Arg het Uncertain

*SFTPA2* chr10-81319089 NM_001098668.4 c.151A>T p.Lys51Ter het Uncertain

**Supplementary table 3.** Other Laboratory features of this patient.

**Laboratory features Levels at diagnosis References values**

CMV-IgG 68 <6AU/ml

EBV-NAIgG positive negative

EBV-CAIgG positive negative

EBV-DNA PCR <400 <400copies/ml

CMV-DNA PCR <400 <400copies/ml

HSV-DNA PCR 1.2*10^4^ <400copies/ml

**Supplementary Figure 1.** Flow cytometry detection of NK cell CD107a analysis of degranulation function of this patient after the HLH.

Patient

HC2

HC1


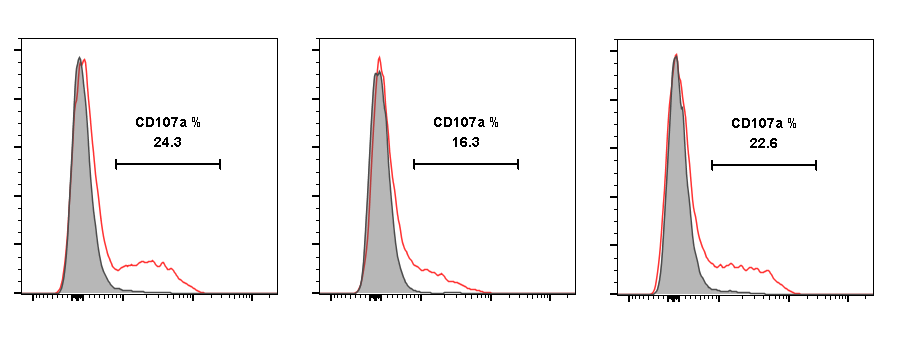


gate in CD56+CD3- cells

CD107a
